# Human Perceptions Mirror Realities of Carnivore Attack Risk for Livestock: Implications for Mitigating Human-Carnivore Conflict

**DOI:** 10.1371/journal.pone.0162685

**Published:** 2016-09-12

**Authors:** Jennifer R. B. Miller, Yadvendradev V. Jhala, Oswald J. Schmitz

**Affiliations:** 1 Yale School of Forestry & Environmental Studies, New Haven, Connecticut, United States of America; 2 Wildlife Institute of India, Dehradun, Uttarakhand, India; Centre for Cellular and Molecular Biology, INDIA

## Abstract

Human-carnivore conflict is challenging to quantify because it is shaped by both the realities and people’s perceptions of carnivore threats. Whether perceptions align with realities can have implications for conflict mitigation: misalignments can lead to heightened and indiscriminant persecution of carnivores whereas alignments can offer deeper insights into human-carnivore interactions. We applied a landscape-scale spatial analysis of livestock killed by tigers and leopards in India to model and map observed attack risk, and surveyed owners of livestock killed by tigers and leopards for their rankings of threats across habitats to map perceived attack risk. Observed tiger risk to livestock was greatest near dense forests and at moderate distances from human activity while leopard risk was greatest near open vegetation. People accurately perceived spatial differences between tiger and leopard hunting patterns, expected greater threat in areas with high values of observed risk for both carnivores. Owners’ perception of threats largely did not depend on environmental conditions surrounding their village (spatial location, dominant land-use or observed carnivore risk). Surveys revealed that owners who previously lost livestock to carnivores used more livestock protection methods than those who had no prior losses, and that owners who had recently lost livestock for the first time expressed greater interest in changing their protection methods than those who experienced prior losses. Our findings suggest that in systems where realities and perceptions of carnivore risk align, conservation programs and policies can optimize conservation outcomes by (1) improving the effectiveness of livestock protection methods and (2) working with owners who have recently lost livestock and are most willing to invest effort in adapting protection strategies to mitigate human-carnivore conflict.

## Introduction

An important challenge in conserving large carnivores in human-dominated landscapes is overcoming human-wildlife conflict arising from people’s real or perceived threats to their livelihoods and personal safety [1,2]. Methods used for reducing potential conflict depend on people accurately perceiving ambient levels of predation threat from different carnivore species to be able to apply protection measures proportionally [3]. However, people’s perceptions of predators are not always in parallel with carnivore behavior because they can be shaped by social and cultural influences, economic pressures, personal values and historical events [4,5]. People’s perceptions can also differ from actual levels of carnivore risk based on the taxonomic identity, physical size or cultural reputation of a carnivore [6]. Collectively, these factors may cause mismatches between real and perceived threats which can create challenges in effectively applying measures of protection against carnivores, such as by implementing mitigation techniques on the wrong species, spatial location or time period, and thus draining resources that would otherwise have helped avoid conflict [7–9].

Stakeholders that can distinguish between threats from carnivores with different hunting behaviors could potentially implement carnivore-specific management strategies to more effectively reduce conflicts. If such stakeholders have suffered livelihood losses from carnivores in the past, they may also be more receptive to investing in mitigation efforts to prevent future conflicts and thus represent a high-priority demographic for human-carnivore coexistence initiatives. Yet it is currently unclear whether people can accurately discriminate predation risks imposed by co-occurring carnivores with different hunting characteristics, and how previous livelihood losses to carnivores affect their efforts to mitigate future human-carnivore conflict.

We investigated these questions by assessing landscape-scale predation threats and protection efforts for livestock from two large carnivores with different hunting characteristics, the tiger (*Panthera tigris*) and the leopard (*Panthera pardus*), in Kanha Tiger Reserve in central India. Tigers hunt primarily in dense forests with low levels of human presence whereas leopards pursue prey in more open, human-dominated landscapes [10–12]. Throughout Asia and many rapidly developing regions worldwide, human population expansion and habitat fragmentation constrict large carnivore ranges into small protected areas juxtaposed against agropastoral communities [13]. The inevitable interactions between these carnivores and livestock result in regular and substantial livelihood losses and retaliations against predators [14,15]. We studied the locations of livestock killed by tigers and leopards to spatially predict attack risk and surveyed owners of depredated livestock for rankings of threat in different land-use types to spatially model their perceived attack risk across the landscape. We also investigated associations between owners’ previous experiences with livestock depredation and their efforts to protect livestock and prevent future attacks. Our study examines the consistency between perceptions of threat with realities and the implications for developing and implementing effective strategies to mitigate human-carnivore conflict.

## Methods

### Ethics statement

Relevant permissions to carry out research were obtained from the Principal Chief Conservator of Forests and Field Director of Kanha Tiger Reserve, Madhya Pradesh Forest Department. In consultation with the Yale University Institutional Review Board, our survey on perceived risk was not considered human subjects research and thus did not require official review. Participants provided verbal informed consent to participate in the study; written consent was not possible since many residents in the study area are illiterate. All people that we asked to participate gave consent, and we recorded consent on the participants’ data sheet during data collection. Any identifying participant information was removed prior to analysis to protect participants’ privacy.

### Study area

The study was conducted in the 2,074 km^2^ core and buffer zones of Kanha Tiger Reserve, Madhya Pradesh, where 93,100 livestock from 178 villages are grazed in and around forests inhabited by approximately 70 tigers and 100 leopards [16,17]. Tigers and leopards kill between 300–600 domestic cattle, buffalo, pigs and goats annually throughout the tiger reserve [17] in roughly equal frequencies [18]. We designated our study area for mapping carnivore attack risk within 4 km of village centers since no livestock were killed beyond this distance [18].

### Observed attack risk

**Fig 1:**
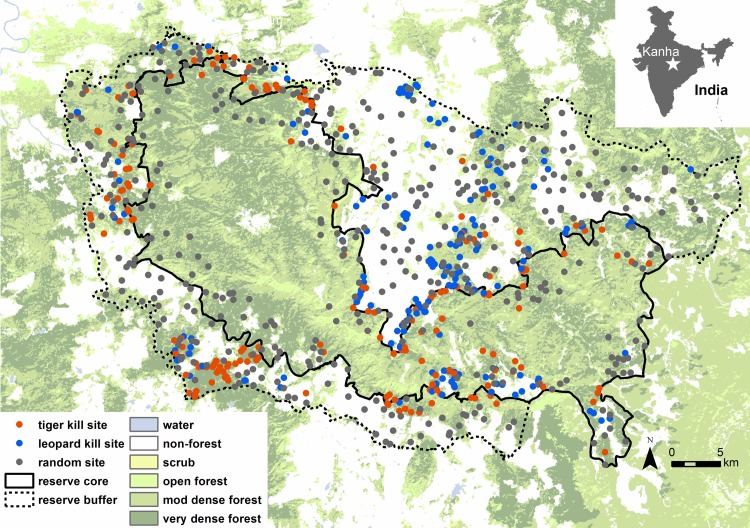
Sampled kill and random sites in Kanha Tiger Reserve, central India. Kill sites for tiger are shown in orange and for leopard are shown in blue, and random sites are shown in grey.

We developed prediction maps of observed attack risk by using sites of carnivore attacks on livestock in the study site to build spatial prediction models of attack risk for tigers and leopards (separate model for each species). Sampling and modeling methods for calculating observed attack risk are described in detail in [11,18]. Models were built as logistic regression ‘use-availability’ resource selection functions based on the values of environmental and anthropogenic predictor variables calculated at 439 random sites and 138 tiger and 128 leopard kill sites where carnivores killed livestock between December 2011 and August 2012 (Fig 1). Risk models were validated for their predictive accuracy against an independent dataset of known livestock kills and used to calculate the relative probabilities of attack risk across the landscape and map tiger and leopard risk in ArcGIS. Because the intercept and coefficients in a use-availability model depend on the distribution of randomly selected samples, model outputs are interpreted as relative but not absolute probabilities of risk. We therefore examined spatial patterns of relative risk distributions (rather than absolute probability values) when comparing results between carnivore species. All statistical analyses were conducted in R (v.2.15.3, R Project Development Team, www.r-project.org) using the MASS, MuMIN and R DAAG packages.

### Perceived attack risk

To assess how livestock owners perceive and respond to attack risk, we interviewed people who reported that their livestock were killed by tigers or leopards during the study period for financial compensation by the local Forest Department. In an effort to prevent livestock owners from retaliating against carnivores, the Forest Department financially compensates owners for livestock killed by wild carnivores. To receive compensation, a livestock owner must locate and report the livestock carcass to the Forest Department within 48 h, and an officer then visits the site to record evidence of the death. Though not all livestock owners choose to report lost livestock (Karanth et al.[19] found that 36% of livestock owners living in the larger landscape who claimed to have lost livestock to carnivores filed for compensation), between 400 and 600 livestock are reported for compensation each year within the tiger reserve [17,18]. During our 9-month study we visited 449 livestock carcasses, which totaled 92% of reported kills in Kanha during that period. Whenever a livestock owner accompanied us to a kill site, we utilized the opportunity to conduct a semi-structured interview with them about perceived risk. We conducted interviews in the form of casual conversations so as not to intimidate participants. We do not expect any biases in attitudes related to carnivores in our sample of livestock owners because most reported livestock in Kanha during 2011–2012 were compensated (91%) quickly (within several weeks) and we were not aware of any social tensions that would have affected compensation reporting during nine months of extensive field visits. We expected that these owners who had recently lost livestock would represent the people in the community most informed about tiger and leopard attack risk, and thus whose perceptions of risk were most closely aligned with model predictions of risk.

We asked owners to rank their perceptions of tiger and leopard attack risk in four major land-use categories: village, agricultural fields, field-forest edge vegetation and forest. Each land-use category was assigned a ranking of 1 (low), 2 (moderately high), 3 (high) or 4 (very high). Not all owners felt capable of assessing risk levels in every land-use category, resulting in unequal sample sizes among land-use categories (*n* = 19–95 for each land-use category and total assigned rankings *n* = 204 for tiger and *n* = 194 for leopard). We also asked owners whether their household had previously lost livestock to wild carnivore). We also asked owners to describe what methods they used to protect livestock and what methods they would use in the future.

Prior to examining the perceptions of owners across the landscape, we conducted a preliminary analysis to explore whether an owner’s perception of carnivore risk was influenced by the environment around their village. We tested whether perceptions of risk were confounded or conflated by the spatial location of an owners’ village and the dominant land cover type surrounding an owners’ village. We ran ordinal logistic regressions with perceived risk in each land-use category as a response variable followed by chi-square tests to indicate model fit. To test the effect of spatial location, we ran regressions with the latitude and longitude of an owner’s village as predictor variables. To test the effect of land cover, we calculated the percent of each land-use type within a 4-km buffer of surveyed village centers and then assessed how perceived risk changed with the abundance by land-use type in each owner’s village. We found that perceptions were not affected by the spatial location of, or dominant land cover type surrounding, an owner’s village (see Results for statistics). These results indicated that an owner’s perception of risk was not specific to his village location or environmental context, removing these as potential confounding factors on our larger analysis of owners across the landscape.

We next examined how owners’ perceive carnivore threats in different types of land-use or, in other words, what environmental conditions carnivores were most likely to attack livestock. We calculated the median risk value for each land-use category and mapped perceived risk using the Forest Survey of India State of the Forests 2009 land-use map. Perceived risk in agricultural fields was mapped as ‘non-forest’, agricultural field-forest edge vegetation as a combination of ‘scrubland’ and ‘open forest’, and forest as a combination of ‘moderately dense forest’ and ‘very dense forest’ categories. Since village areas were not featured in the land-use map, we used heads-up digitization with Google Earth satellite imagery from 2007–2013 to outline village areas (defined as clusters of houses) and mapped the perceived risk in village to this land-use category. We quantified the variation in owners’ responses by calculating the proportion of all responses that differed from the median perceived risk in each land-use category.

We expected that previously losing livestock to carnivores would strengthen the accuracy of a person’s understanding of risk (i.e. that perceived attack risk would more closely resemble model predictions of observed attack risk) than owners who had not previously experienced a carnivore attack. To test whether owners’ risk perceptions changed if their household’s livestock had been previously depredated, we ran one-way ANOVAs on the perceived risk for each land-use category. We also expected that owners who had previously lost livestock to carnivores would use more protection methods and that owners who lost livestock for the first time might be more open to trying new methods for protecting livestock. We ran one-way ANOVAs to test the effect of a previous attack on owners’ past and future use of livestock protection methods.

### Comparing observed and perceived attack risk

We explored spatial associations between model predictions of observed carnivore attack risk on livestock and owners’ perceptions of attack risk. We calculated the mean observed risk within 500 m of surveyed village centers and tested its role in predicting the perceived risk of owners from each village using ordinal logistic regression with chi-square tests to indicate model fit. To compare the spatial distributions of observed and perceived risk, we sampled the observed risk and the median perceived risk in each pixel across the landscape and used boxplots to examine differences across the study area. We ran Kruskal Wallis tests with Bonferroni correction followed by Dunn’s multiple comparisons post-hoc tests to test for differences in observed risk between perceived risk levels.

## Results

### Observed attack risk

**Fig 2:**
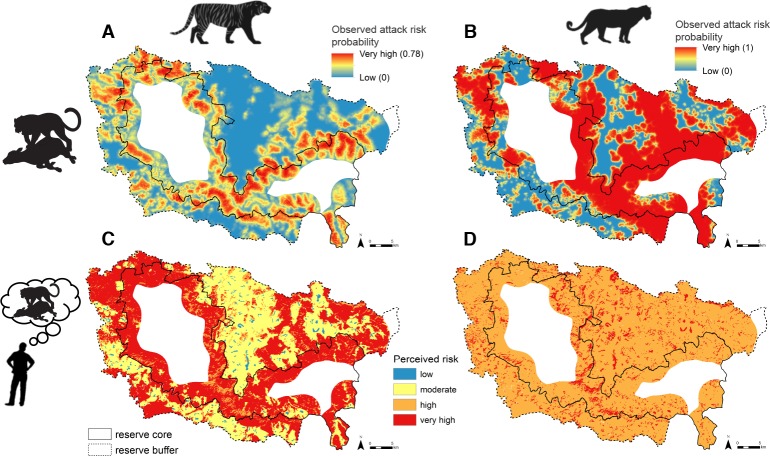
**Maps showing model predictions of observed attack risk (top, A and B) and livestock owners’ perceptions of attack risk (bottom, C and D) for tiger (left, A and C) and leopard (right, B and D) on livestock.** Maps represent relative probabilities of risk; absolute magnitudes of risk are not directly comparable.

Models of attack risk revealed that tiger and leopard kills were associated with unique sets of landscape features (Table 1), forming distinct spatial distributions of predation risk (Fig 2A and 2B). Livestock were most vulnerable to tigers near moderately and very dense forest (such as near the core zone of the park) and at intermediate distances from roads, villages and scrubland (Table 1). The relative kill probability showed a threshold relationship to the distance to road, village and scrubland, with risk peaking 1.2 km from roads, 1.1 km from villages and 8.0 km from scrublands (S1 Fig). Relative predicted tiger risk levels varied from 0–0.77 across the landscape and predominantly ranged within low and intermediate levels, with rare patches of very high risk located directly within the park core zone boundary (Fig 2A). Leopards were more likely to kill livestock near scrubland and open and moderately dense forest and far from water (Table 1). Leopard relative risk ranged from 0–1.00 and was very high across most of the landscape, including human-dominated areas with villages and agricultural fields (S2 Fig). All variables included in each carnivore model contributed strongly to model predictions and had relative importance values greater than 0.40 (Table 1). Tiger and leopard models correctly identified 63% (44 of 70) and 85% (61 of 72) of known validation kills, respectively, which is significantly greater than would be expected by random chance (*P* = 0.001 for tiger; *P* < 0.001 for leopard; S3 Fig).

**Table 1:** Final model output for tiger and leopard observed attack risk for livestock.

Variable	Tiger				Leopard			
	*β*	SE	*P-*value	Relative importance	*β*	SE	*P-*value	Relative importance
Intercept	-2.58	0.71	0.00	NA	-1.22	0.40	2.4E-03	NA
Distance to scrubland	3.6E-04	1.3E-04	6.8E-03	0.95	1.2E-04	1.4E-04	0.38	0.50
Distance to scrubland^2^	-2.2E-08	8.1E-09	6.5E-03	0.97	-1.5E-08	8.1E-09	0.07	0.95
Distance to moderately dense forest	-1.5E-03	1.2E-03	0.21	0.44	-1.9E-03	6.4E-04	3.1E-03	0.98
Distance to open forest					-8.0E-04	5.0E-04	0.11	0.60
Distance to water					2.4E-04	6.1E-05	8.3E-05	1.00
Distance to village	8.9E-04	5.5E-04	0.11	0.73				
Distance to village^2^	-3.4E-07	2.0E-07	8.6E-02	0.85				
Distance to road	3.0E-03	6.6E-04	6.1E-06	1.00				
Distance to road^2^	-1.2E-06	3.3E-07	2.6E-04	1.00				
Distance to core zone	-1.5E-04	5.4E-05	5.7E-03	0.99				
Distance to very dense forest	-3.5E-03	8.7E-04	6.4E-05	1.00				

*β*, coefficients; SE, standard error.

Blank cells indicate that the variable was not included in the model.

‘NA’ indicates statistic is not applicable.

### Perceived attack risk

**Fig 3:**
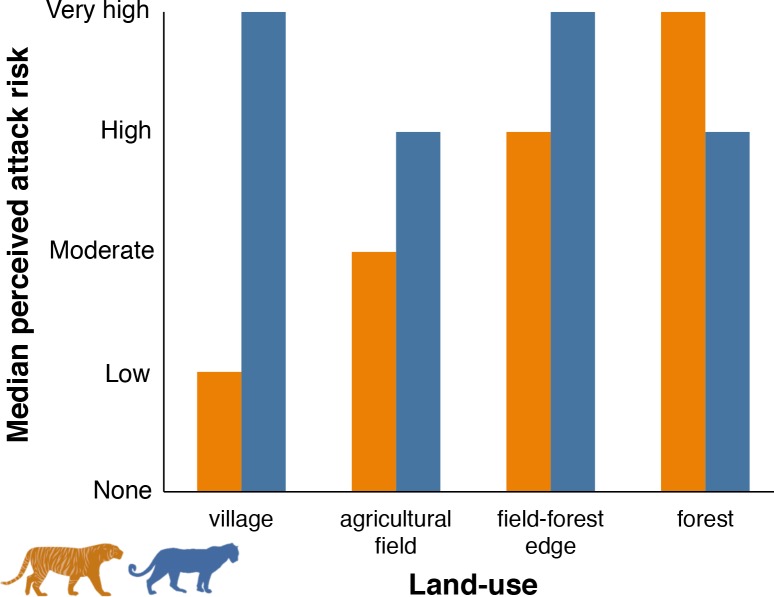
Livestock owners median perceived attack risk from tigers and leopards in different land-use categories. Tiger risk is shown in orange; leopard risk is shown in blue. Sample size: *n*_village-tiger_ = 19; *n*_village-leopard_ = 44; *n*_field-tiger_ = 39; *n*_field-leopard_ = 50; *n*_edge-tiger_ = 51; *n*_edge-leopard_ = 40; *n*_forest-tiger_ = 95; *n*_forest-leopard_ = 60.

**Fig 4:**
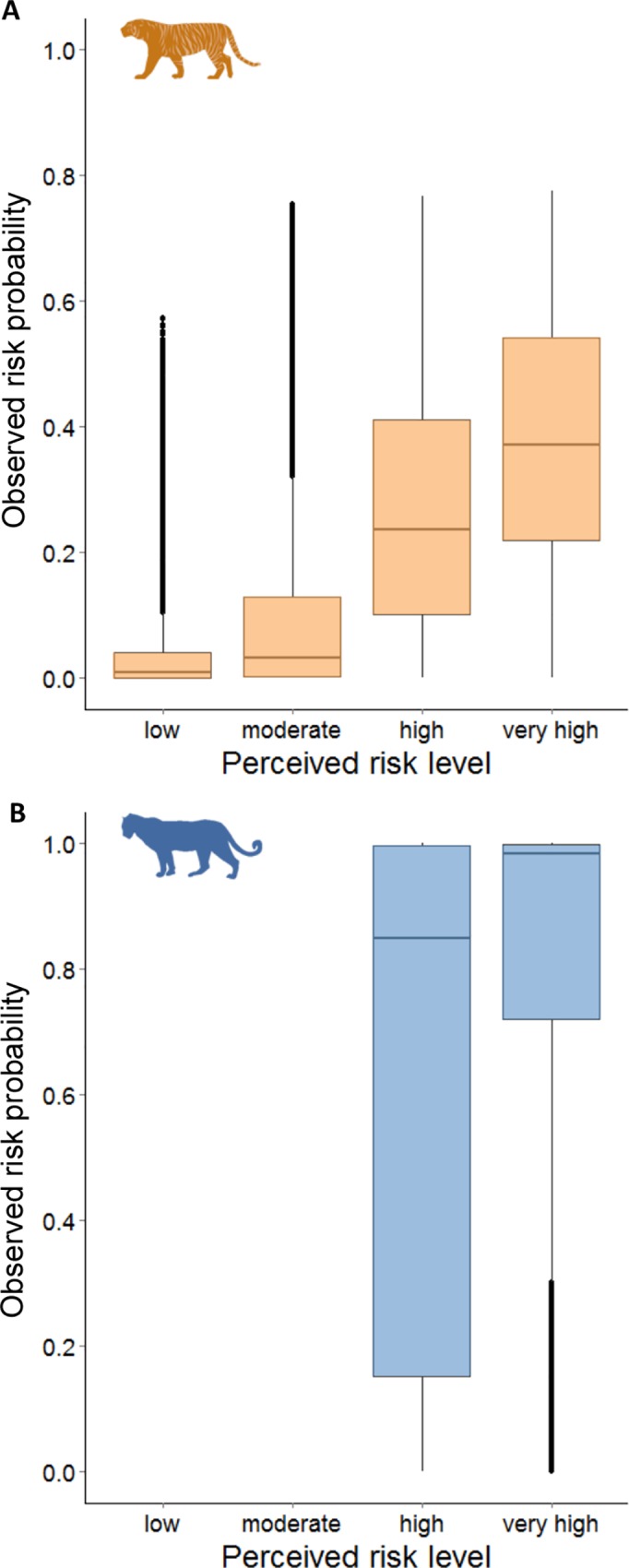
**Relationship between perceived and observed risk from tiger (A, orange) and leopard (B, blue) on livestock in each pixel across the Kanha landscape.** Kruskal Wallis tests with Dunn’s post-hoc tests indicated that all perceived risk levels within each plot significantly differed from one another (*P* < 0.001).

**Fig 5:**
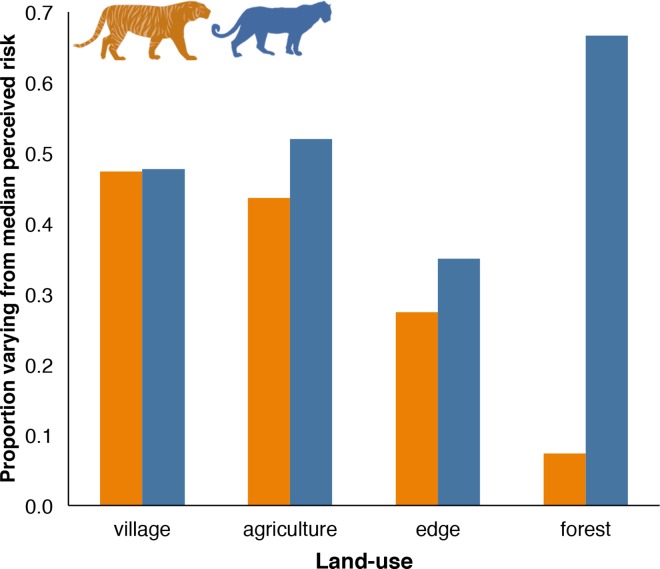
Variation in individual owners’ perceived risk within each land-use category. Variation was calculated as the proportion of owners that deviated from the median perceived risk level. Larger values represent greater variation between owners’ responses and lower overall alignment in owners' perceptions of risk. Perceived risk from tigers is shown in orange and from leopards is shown in blue.

Owners’ perceptions of tiger and leopard risk closely mirrored spatial distributions of observed risk from both carnivores across the landscape. Owners perceived the lowest risks from tigers in village and gradually increasing risks across land-use categories with denser forest and less human infrastructure (Fig 3), a gradient that matched the spatial pattern of observed tiger attack risk (Fig 2A and 2C). Livestock owners perceived intense risks from leopard in all land-use categories: agricultural fields and forest were both ranked as high risk and villages and agricultural field-forest edge were both ranked as very high risk (Fig 3). This ranking resembled the extensive swaths of elevated observed attack risk from leopard across the region (Fig 2B and 2D). In comparing observed and perceived risk values at points across the landscape, we found that observed attack risk significantly differed between perceived risk levels and increased with higher levels of perceived risk for both tiger (χ^2^(3) = 142,342, *P* < 0.001; Dunn’s test *P* < 0.001) and leopard (χ^2^(3) = 64,029, *P* < 0.001; Dunn’s test *P* < 0.001; Fig 4). Boxplots for leopards within each perceived risk level were skewed towards lower values of observed attack probability (Fig 4B), which was explained by the greater variation in owners’ responses for leopard than tiger risk (Fig 5).

Owners’ perceptions of leopard risk were unaffected by the spatial location (χ^2^ < 0.05; S1 Table), amount or type of land-use (χ^2^ < 0.05; S2 Table) or observed attack risk (χ^2^ < 0.05; S3 Table) in or around their villages. Perceptions of tiger threats were not influenced by village spatial location (χ^2^ < 0.05; S1 Table), the amount of village, agricultural field or field-forest edge (χ^2^ < 0.05; S2 Table) or the observed risk in these land-use categories (χ^2^ < 0.05; S3 Table). However, owners living in more southern and forested areas tended to rank tiger risk as high in forest and owners living in areas with higher observed risk ranked threats from tigers as lower in forest (χ^2^ = 1.00 for all tests).

### Livestock protection methods

All 112 owners we spoke with were men, as no women claimed livestock compensation for their households during the sampling period. Across all owners, 82% used enclosures to confine livestock at night, and 65% and 20% used family herders or hired non-family herders to supervise grazing livestock, respectively. Nearly half of owners (45%) had previously lost livestock to tigers or leopards. Previous losses did not affect owners’ perceptions of tiger or leopard threats in any land-use category (all ANOVAs: *P* > 0.05; S4 Table) but did affect owners’ efforts to protect livestock. At the time of the survey, significantly more owners whose livestock had been attacked in the past (74%) used family members to guard grazing livestock than did owners who lost livestock for the first time (54%; F_1,93_ = 4.389, *P* = 0.039), while all other protection methods (livestock enclosures and hired herders) were used equally (enclosures: F_1,93_ = 1.267, *P* = 0.263; herders: F_1,93_ = 1.532, *P* = 0.219). When asked what protection efforts they would change in the future, significantly more owners who lost livestock for the first time (21%) showed interest in changing grazing areas compared to owners with previous losses (10%; F_1,93_ = 5.176, *P* = 0.025); all other future changes did not differ by previous experience (all ANOVAs: *P* > 0.05; S5 Table). Across all owners, 32% of all owners said that in the future they would tie livestock near the house at night, 22% would change the grazing area, 21% would strengthen night enclosures for livestock, 20% would hire non-family herders, 12% would use family herders and 11% would generally stay more alert when watching livestock.

## Discussion

Our study offers a unique spatial perspective on how realities and perceptions of carnivore threats compare, with direct implications for on-the-ground management and conservation. Owners with livestock killed by tigers and leopards accurately perceived the hunting patterns and spatial distributions of tiger and leopard attacks on livestock. Model predictions of risk based on depredations showed that tiger and leopard livestock kills were associated with distinct suites of habitat features, with tigers more likely to kill domestic animals near dense vegetation and away from human infrastructure and leopards tending to kill livestock close to open vegetation. Owners sensed these different hunting characteristics so precisely that the mapped distributions of perceived risks closely matched the spatial gradient of modeled observed risk for both tigers and leopards. Previous experience with depredation did not affect owners’ perceptions of risk, suggesting that risk perceptions are strongly held concepts that may not change over time or with increased direct or indirect interactions with carnivores. The close alignment between perceptions and realities of tiger and leopard threats contrasts with other carnivore species, such as snow leopards and wolves in northern India, where human perceptions substantially differ from the realities of livestock depredation [9]. This alignment may indicate an opportunity to implement adaptable carnivore-specific strategies that more effectively mitigate human-carnivore conflict.

For instance, the fact that owners distinguished distinct hunting behaviors and spatial patterns of risk for tigers and leopards suggests that people could adapt livestock management and protection based on the behavioral traits of each carnivore. Drawing from animal behavioral ecology, wild prey (here akin to people and their livestock) predictably modify their escape tactics in response to a predator’s hunting mode and habitat domain, adapting movement, vigilance and foraging behaviors to optimize survival across the landscape [20–22]. Prey tend to be highly vigilant around stalking ambush predators but suffer greater mortality from active courser predators, and prey shift their habitat use (space) to avoid narrow-domain predators whereas they adjust habitat or activity (space or time) to avoid broad-domain predators [23]. Conservation practitioners and local stakeholders could apply this framework to adjust human-carnivore mitigation strategies based on the ecological role of the carnivores in their system. For example, tigers and leopards utilize similar stalking hunting modes but distinct habitat domains, with tigers limited to dense forests away from human presence and leopards inhabiting a broader range of open vegetation and human-dominated areas. Attacks from carnivores like tigers (narrow domain) could likely best be avoided by shifting livestock away from tiger-inhabited areas, such as the forested protected area core zone. To avoid carnivores like leopards (broader domain), owners would instead need to adjust livestock activity or protective infrastructure across the landscape, such as by changing the timing of grazing patterns or strengthening overnight livestock enclosures. Protection strategies will be most effective if they defend against the carnivore most likely to attack in a given area.

In central India and many landscapes worldwide, livestock owners and park managers simultaneously apply multiple strategies to protect livestock from a community of predator species [3,19]. This approach is advantageous in areas where multiple predators present high threats in the same areas or where risks are challenging for stakeholders to distinguish. Yet where appropriate, the risk probability of a carnivore attack could serve as a useful metric for strategically implementing protection strategies to potentially reduce costs and strengthen the effectiveness of techniques. Previous studies have also encouraged carnivore-specific strategies for reducing livestock depredation [8,24]. For example, livestock managers could adjust protection strategies relative to the likelihood of attack from different carnivore species, and park management could use the risks from different predators as a basis for incentivizing livestock owners to implement adaptive responses. Such adaptive mitigation strategies would be especially effective when implemented among stakeholders that accurately perceive the realized distribution of attack risk from carnivores.

The association between owners’ previous experience of losing livestock to carnivores and their efforts to protect livestock offers insight into an important factor driving behavioral change that could improve the effectiveness of conservation programs and policies aimed at reducing human-carnivore conflict. Owners who had previously lost livestock invested extra effort into protecting their animals by sending family members to herd grazing livestock (in addition to hiring herders and using enclosures like owners who had no previous experience with depredation). Sending a family member to accompany livestock–which could jeopardize the person’s safety [25]–represents a significant personal investment in livestock protection that appears to have stemmed from a heightened awareness of carnivore threats due to first-hand experience with the previous attack on their livestock. Likewise, owners who lost livestock for the first time at the time of the interview expressed greater interest in more rigorously protecting livestock in the future by changing their livestock grazing areas. These results collectively demonstrate that the experience of losing livestock to carnivores motivated owners to use more extensive protection methods and suggest that owners who have lost livestock *for the first time* are most willing to change. Programs and policies aimed at reducing livestock losses to carnivores should therefore prioritize owners who have recently lost livestock for education and subsidies (e.g. reduced fees for work plans and materials to build predator-proof enclosures) since these people are likely to be more receptive to devoting time and resources to improving their livestock protection strategies. This information should be combined with other known factors associated with more positive attitudes towards carnivores, such as higher level of education, younger age, larger village size, greater agricultural production, smaller holdings of large-bodied livestock stock, greater diversity of income sources and greater benefits associated with carnivore presence (e.g. tourism, ecosystem health) [4,26,27].

Our results also showed that owners’ perception of threats largely did not depend on the dominant land-use or frequency of observed kills near their villages. However, people living in southern and forested areas perceived greater tiger risk in forest and owners living in areas with a higher observed attack risk perceived lower risk in forest. Because dense forest is a strong indicator of kills, owners living near forests likely observe more tiger attacks in forest than people living in more open land types and thus consistently associate forest with high tiger risk. In contrast, owners living in areas with a high frequency of observed kills, regardless of the surrounding land type, may observe tigers killing livestock over a greater variety of land types and therefore less frequently associate forest explicitly with high risk. This suggests that conservation practitioners may need to adapt strategies for reducing livestock depredation in different areas based on local perceptions of risk. In addition, owners showed greater uncertainty in their perceptions of leopard (than tiger) risk across all land-use categories, possibly due to the extensive distribution of high leopard observed attack risk across the landscape. Because leopards utilize a broader variety of habitats than tigers, hunting in both forested and human-dominated areas [12], their selection of specific habitats when hunting livestock may be too varied for people to precisely decipher through observation.

Communities in southern Asia are renowned for being some of the most tolerant towards carnivore conservation globally [28,29]. This likely stems from ethical and cultural values shaped by religion, since Hinduism and Buddhism, dominant in many areas, encourage equality between human and non-human life forms and respect and compassion for the natural world [30,31]. This positive attitude towards nature, and carnivores by extension, has contributed to the lower rates of historical persecution against carnivores than in Europe and North America, for example [1,32,33]. However, it is not yet clear whether or how attitude (commonly oversimplified in research as ‘positive’ or ‘negative’; [34]) affects accuracy in understanding about carnivore ecology or perceptions of threats (e.g. where carnivores attack). By comparing two South Asian communities that both traditionally respect animals–our results from central India (where observed and perceived risk of tigers and leopards align) versus those from central Nepal (where observed and perceived risk of snow leopards and wolves differ [9])–reveals that perceptions about carnivore threats can differ by region and carnivore species. Although local people may support carnivore conservation, their ability to appropriately implement methods for preventing livestock losses to carnivores will be impaired if their perceptions of where carnivores attack do not reflect realities [4]. Additional research is needed to better refine our understanding of how perceptions of threats from the *same* carnivore species vary across landscapes and which factors shape people’s spatial understanding of–and responses to–carnivore threats. Integrating spatial, ecological and social perspectives will be key to formulating a comprehensive framework for resolving human-wildlife conflict.

Our sample for measuring perceived risk was limited to owners of livestock killed by tigers and leopard and we recognize that results do not account for the perceptions of all livestock owners or all people living in the study area. Because the villages in Kanha are small, ranging from 100–2,000 people per village (Kanha Tiger Reserve Forest Department 2012), and people openly share stories and opinions about tigers and leopards (J. Miller, personal observation), we do not expect that the perceptions of owners with depredated livestock differed substantially from other people in the community. However, if the process of locating livestock carcasses in the landscape refined owners’ spatial understanding of carnivore risk, we would expect the people we interviewed to reflect the most accurate perceptions of risk in the study area. Furthermore, we recognize that people’s perceptions of carnivore risk may not be homogeneous across a single land-cover type yet also acknowledge that a coarse-level of understanding can be valuable for informing further investigation. We encourage future studies to survey the broader community in order to incorporate more diverse perspectives into conservation decision-making.

## Conclusions

Our findings provide spatial insight into the relationships between humans and carnivores by showing that people who have lost livestock to carnivores can accurately estimate threats from carnivores at a landscape scale. This has significant implications for conservation because programs and policies aimed at reducing human-carnivore conflict can channel efforts towards improving the effectiveness of livestock protection methods rather than educating stakeholders on where to implement them. Our study also reveals that livestock owners are particularly motivated to invest in livestock protection methods if they or their families previously lost livestock to carnivores, especially in the recent past. These people should be prioritized for receiving training or financial subsidies to improve livestock protection methods in order to optimize conservation outcomes.

Because understanding informs action, the spatial alignment between perceived and observed carnivore risk suggests that people take precautionary measures to protect livestock relative to the level of risk at a given site and their first-hand experience with losing livestock to carnivores. The fact that livestock depredation continues to occur underscores the complexities that link people’s perceptions of risk with their motivation to reduce threats, as well as the inherent challenges of preventing carnivore attacks. Comparing the realities and perceptions of carnivore threats represents one important step towards strategically enhancing efforts for mitigating human-carnivore conflict in ways that facilitate coexistence with large carnivores.

## Supporting Information

S1 Fig Tiger observed kill risk probability for each variable. (TIF)

S2 Fig Leopard observed kill risk probability for each variable. (TIF)

S3 Fig Model validation results for observed attack risk. (TIF)

S1 Table Ordinal logistic regression results testing effect of village location on perceived risk. (DOCX)

S2 Table Ordinal logistic regression results testing effect of land-use near villages on perceived risk. (DOCX)

S3 Table Ordinal logistic regression results testing effect of observed risk near villages on perceived risk. (DOCX)

S4 Table ANOVA results for effects of previous experience with livestock depredation on perceived risk. (DOCX)

S5 Table ANOVA results for effects of previous experience with livestock depredation on use of livestock protection methods. (DOCX)

## References

[1] Ripple WJ, Estes JA, Beschta RL, Wilmers CC, Ritchie EG, Hebblewhite M, et al Status and ecological effects of the world’s largest carnivores. Science 2014;343:1–11. 10.1126/science.1241484 24408439

[2] Woodroffe R, Thirgood S, Rabinowitz A. People and Wildlife: Conflict or Coexistence? Cambridge: Cambridge University Press; 2005.

[3] Linnell JDC, Odden J, Mertens A. Mitigation methods for conflicts associated with carnivore depredation on livestock In: Boitani L, Powell RA, editors. Carnivore Ecology and Conservation: Handbook of Techniques. Oxford: Oxford University Press; 2012 pp. 314–32.

[4] Dickman AJ. Complexities of conflict: the importance of considering social factors for effectively resolving human-wildlife conflict. Anim. Conserv. 2010;13:458–66.

[5] Bruskotter JT, Wilson RS. Determining where the wild things will be: using psychological theory to find tolerance for large carnivores. Conserv. Lett. 2014;7:158–65.

[6] Kellert SR. Public perceptions of predators, particularly the wolf and coyote. Biol. Conserv. 1985;31:167–89.

[7] Mishra C. Livestock depredation by large carnivores in the Indian trans-Himalaya: conflict perceptions and conservation prospects. Environ. Conserv. 1997;24:338–43.

[8] Ogada MO, Woodroffe R, Oguge NO, Frank LG. Limiting depredation by African carnivores: the role of livestock husbandry. Conserv. Biol. 2003;17:1521–30.

[9] Suryawanshi KR, Bhatnagar YV, Redpath S, Mishra C. People, predators and perceptions: patterns of livestock depredation by snow leopards and wolves. Pettorelli N, editor. J. Appl. Ecol. 2013;50:550–60.

[10] Karanth KU, Sunquist ME. Behavioural correlates of predation by tiger (Panthera tigris), leopard (Panthera pardus) and dhole (Cuon alpinus) in Nagarahole, India. J. Zool. 2000;250:255–65.

[11] Miller JRB, Jhala Y V., Jena J, Schmitz OJ. Landscape-scale accessibility of livestock to tigers: implications of spatial grain for modeling predation risk to mitigate human-carnivore conflict. Ecol. Evol. 2015;5:1354–67. 10.1002/ece3.1440 25859339 PMC4377277

[12] Athreya VR, Odden M, Linnell JDC, Krishnaswamy J, Karanth KU. Big cats in our backyards: persistence of large carnivores in a human dominated landscape in India. PLOS ONE. 2013;8:e57872 10.1371/journal.pone.0057872 23483933 PMC3590292

[13] Sharma S, Dutta T, Maldonado JE, Wood TC, Panwar HS, Seidensticker J. Forest corridors maintain historical gene flow in a tiger metapopulation in the highlands of central India. Proc. R. Soc. B. 2013;280:20131506 10.1098/rspb.2013.1506 23902910 PMC3735263

[14] Treves A, Karanth KU. Human-carnivore conflict and perspectives on carnivore management worldwide. Conserv. Biol. 2003;17:1491–9.

[15] Karanth KK, Naughton-Treves L, DeFries RS, Gopalaswamy AM. Living with wildlife and mitigating conflicts around three Indian protected areas. Environ. Manage. 2013;52:1320–32. 10.1007/s00267-013-0162-1 24026255

[16] Jhala Y V., Qureshi Q, Vettakevan J, Bohra J, Yumnam B, Kumar U, et al. Spatial and population ecology of tiger co-predator and their prey in Kanha tiger reserve. Progress report 2005–2013. Dehradun, Delhi and Mandla; 2014.

[17] Negi H, Shukla R. Tiger Conservation Plan for Kanha Tiger Reserve (2012–2022). Mandla; 2012.

[18] Miller JRB, Jhala Y V., Jena J. Livestock losses and hotspots of attack from tigers and leopards in Kanha Tiger Reserve, Central India. Reg. Environ. Chang. 2016;16:17–29.

[19] Karanth KK, Gopalaswamy AM, DeFries RS, Ballal N. Assessing patterns of human-wildlife conflicts and compensation around a central Indian protected area. PLOS ONE 2012;7:e50433 10.1371/journal.pone.0050433 23227173 PMC3515612

[20] Laundré JW, Hernandez L, Ripple WJ. The landscape of fear: ecological implications of being afraid. Open Ecol. J. 2010;3:1–7.

[21] Miller JRB, Ament JA, Schmitz OJ. Fear on the move: predator hunting mode predicts variation in prey mortality and plasticity in prey spatial response. J. Anim. Ecol. 2014;83:214–22. 10.1111/1365-2656.12111 24028410

[22] Preisser EL, Bolnick DI. The many faces of fear: comparing the pathways and impacts of nonconsumptive predator effects on prey populations. PLOS ONE. 2008;3:e2465 10.1371/journal.pone.0002465 18560575 PMC2409076

[23] Schmitz OJ, Krivan V, Ovadia O. Trophic cascades: the primacy of trait-mediated indirect interactions. Ecol. Lett. 2004;7:153–63.

[24] Kolowski J, Holekamp KEK. Spatial, temporal, and physical characteristics of livestock depredations by large carnivores along a Kenyan reserve border. Biol. Conserv;128:529–41.

[25] Dhanwatey HS, Crawford JC, Abade L a. S, Dhanwatey PH, Nielsen CK, Sillero-Zubiri C. Large carnivore attacks on humans in central India: a case study from the Tadoba-Andhari Tiger Reserve. Oryx 2013;47:221–7.

[26] Suryawanshi KR, Bhatia S, Bhatnagar YV, Redpath S, Mishra C. Multiscale factors affecting human attitudes toward snow leopards and wolves. Conserv. Biol. 2014;28:1657–66. 10.1111/cobi.12320 25039397

[27] Carter NH, Riley SJ, Liu J. Utility of a psychological framework for carnivore conservation. Oryx. 2012;46:525–35.

[28] Bagchi S, Mishra C. Living with large carnivores: predation on livestock by the snow leopard (Uncia uncia). J. Zool. 2006;268:217–24.

[29] Karanth KK, Nichols JD, Karanth KU, Hines JE, Christensen NL. The shrinking ark: patterns of large mammal extinctions in India. Proc. R. Soc. B 2010 10.1098/rspb.2010.0171 PMC2880105 20219736

[30] Sessions G. The Deep Ecology Movement: a review. Environ. Rev. 1987;11:105–25.

[31] Negi CS. Religion and biodiversity conservation: not a mere analogy. Int. J. Biodivers. Sci. Ecosyst. Serv. Manag. 2005;1:85–96.

[32] Treves A, Chapron G, López-Bao J V., Shoemaker C, Goeckner AR, Bruskotter JT. Predators and the public trust. Biol. Rev. 2015 10.1111/brv.12227 PMC5245106 26526656

[33] Chapron G, Kaczensky P, Linnell JDC, von Arx M, Huber D, Andren H, et al Recovery of large carnivores in Europe’s modern human-dominated landscapes. Science 2014;346:1517–9. 10.1126/science.1257553 25525247

[34] Treves A, Wallace RB, Morales A. Co-managing human-wildlife conflicts: a review. Hum. Dimens. Wildl. 2006;11:383–96.

