# Supplementary figures and images for: Human Perceptions Mirror Realities of Carnivore Attack Risk for Livestock: Implications for Mitigating Human-Carnivore Conflict
Source: PLoS One. 2016 Sep 12;11(9):e0162685. doi: 10.1371/journal.pone.0162685 (PMC5019480; doi:10.1371/journal.pone.0162685)

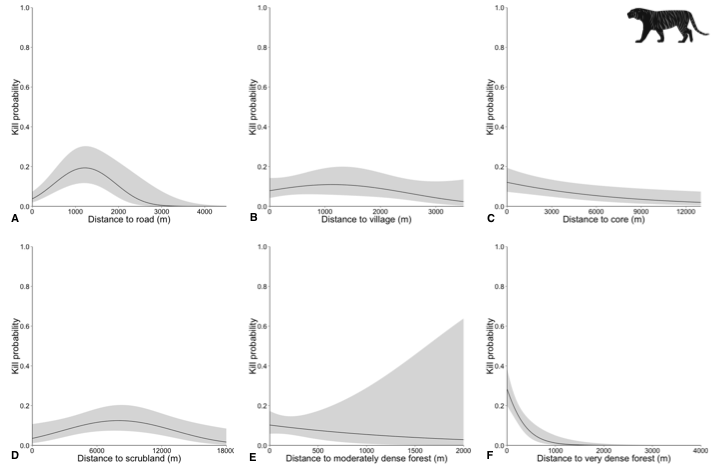

Supplement: S1 Fig — (TIF) [file pone.0162685.s001.tif]

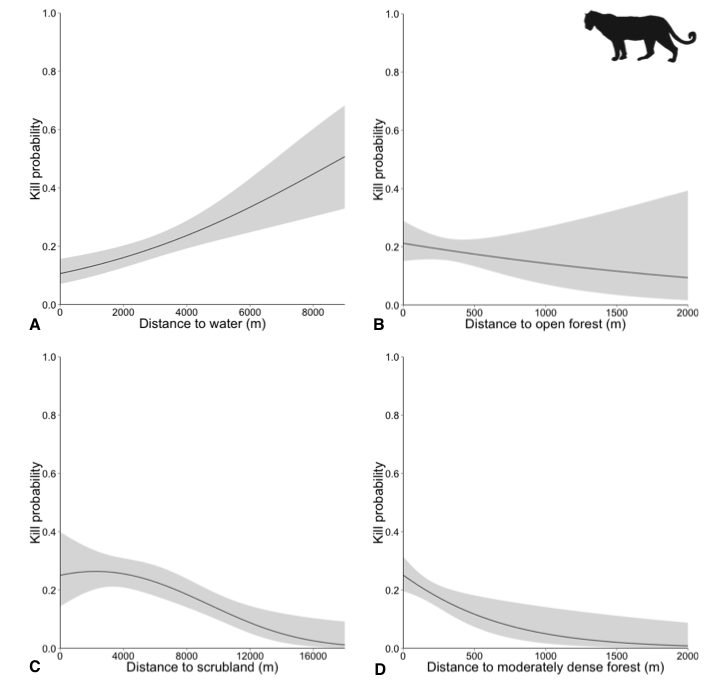

Supplement: S2 Fig — (TIF) [file pone.0162685.s002.tif]

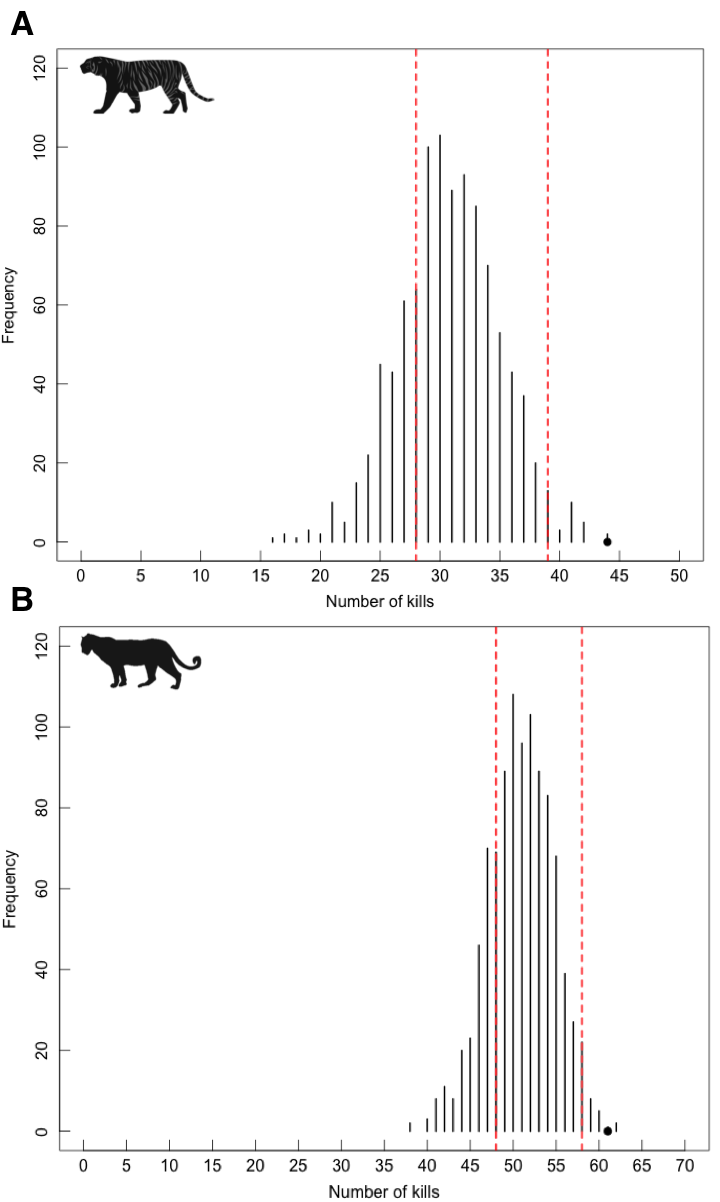

Supplement: S3 Fig — (TIF) [file pone.0162685.s003.tif]
