# Supplementary material for: Human Perceptions Mirror Realities of Carnivore Attack Risk for Livestock: Implications for Mitigating Human-Carnivore Conflict
Source: PLoS One. 2016 Sep 12;11(9):e0162685. doi: 10.1371/journal.pone.0162685 (PMC5019480; doi:10.1371/journal.pone.0162685)
Supplement: S1 Table — (DOCX) [file pone.0162685.s004.docx]

**S1 Table.** Statistics from ordinal logistic regression models testing whether village location influenced owners' perceptions of carnivore risk. Statistics include the coefficient or intercept value, standard error (SE), t-value, *P*-value and the model residual deviance, Akaike Information Criterion (AIC) and chai-square p-value (χ^2^). χ^2^ > 0.05 indicate strong model fit.

| Carnivore species | Land-use | Statistic | Value | SE | t-value | *P*-value | Residual deviance | AIC | χ^2^ |
| --- | --- | --- | --- | --- | --- | --- | --- | --- | --- |
| Tiger | village | intercept 1\|2 | 773.149 | 0.027 | 2.85E+04 | <0.0001 | 35.44 | 45.44 | 1.27E-03 |
|  |  | intercept 2\|3 | 776.114 | 0.730 | 7.80E-01 | <0.0001 |  |  |  |
|  |  | intercept 3\|4 | 776.391 | 1.200 | 2.63E+00 | <0.0001 |  |  |  |
|  |  | coefficient lat | 2.196 | 4.821 | 4.56E-01 | 0.6487 |  |  |  |
|  |  | coefficient long | 9.014 | 1.329 | 6.78E+00 | <0.0001 |  |  |  |
|  | agricultural field | intercept 1\|2 | 230.369 | 0.035 | 6.62E+03 | <0.0001 | 67.30 | 75.30 | 8.34E-04 |
|  |  | intercept 2\|3 | 232.642 | 0.587 | 3.96E+02 | <0.0001 |  |  |  |
|  |  | coefficient lat | 3.627 | 3.739 | 9.70E-01 | 0.3321 |  |  |  |
|  |  | coefficient long | 1.887 | 1.027 | 1.84E+00 | 0.0663 |  |  |  |
|  | agricultural field-forest edge | intercept 1\|2 | 349.790 | 0.020 | 1.72E+04 | <0.0001 | 67.54 | 75.54 | 2.64E-02 |
|  |  | intercept 2\|3 | 354.538 | 0.818 | 4.33E+02 | <0.0001 |  |  |  |
|  |  | coefficient lat | 11.350 | 4.964 | 2.286493e | 0.0222 |  |  |  |
|  |  | coefficient long | 1.226 | 1.363 | 8.99E-01 | 0.3685 |  |  |  |
|  | forest | intercept 1\|2 | -33.642 | 0.015 | -2.18E+03 | <0.0001 | 57.98 | 65.98 | 9.97E-01 |
|  |  | intercept 2\|4 | -32.332 | 0.612 | -5.29E+01 | <0.0001 |  |  |  |
|  |  | coefficient lat | -3.195 | 5.108 | -6.25E-01 | 0.5317 |  |  |  |
|  |  | coefficient long | 0.430 | 1.403 | 3.07E-01 | 0.7591 |  |  |  |
| Leopard | village | intercept 1\|2 | 919.189 | 0.015 | 5.95E+04 | <0.0001 | 95.00 | 105.00 | 1.42E-06 |
|  |  | intercept 2\|3 | 919.819 | 0.242 | 3.79E+03 | <0.0001 |  |  |  |
|  |  | intercept 3\|4 | 920.066 | 0.287 | 3.21E+03 | <0.0001 |  |  |  |
|  |  | coefficient lat | 5.958 | 5.958 | 1.59E+00 | 0.1208 |  |  |  |
|  |  | coefficient long | 9.742 | 1.031 | 9.45E+00 | <0.0001 |  |  |  |
|  | agricultural field | intercept 1\|2 | 80.691 | 0.025 | 3.24E+03 | <0.0001 | 112.28 | 122.28 | 1.13E-07 |
|  |  | intercept 2\|3 | 82.806 | 0.372 | 2.23E+02 | <0.0001 |  |  |  |
|  |  | intercept 3\|4 | 85.808 | 1.051 | 8.17E+01 | <0.0001 |  |  |  |
|  |  | coefficient lat | 3.484 | 3.188 | 1.093045e | 0.2744 |  |  |  |
|  |  | coefficient long | 0.055 | 0.877 | 6.33E-02 | 0.9495 |  |  |  |
|  | agricultural field-forest edge | intercept 1\|2 | -255.212 | 0.025 | -1.02E+04 | <0.0001 | 63.67 | 71.67 | 3.00E-03 |
|  |  | intercept 2\|3 | -253.201 | 0.586 | -4.32E+02 | <0.0001 |  |  |  |
|  |  | coefficient lat | 5.123 | 4.427 | 1.16E+00 | 0.2471 |  |  |  |
|  |  | coefficient long | -4.574 | 1.217 | -3.76E+00 | 0.0002 |  |  |  |
|  | forest | intercept 1\|2 | -136.727 | 0.009 | -1.48E+04 | <0.0001 | 144.55 | 154.55 | 5.61E-10 |
|  |  | intercept 2\|3 | -135.262 | 0.292 | -4.63E+02 | <0.0001 |  |  |  |
|  |  | intercept 3\|4 | -133.809 | 0.523 | -2.56E+02 | <0.0001 |  |  |  |
|  |  | coefficient lat | -1.015 | 2.926 | -3.47E-01 | 0.7286 |  |  |  |
|  |  | coefficient long | -1.410 | 0.805 | -1.75E+00 | 0.0797 |  |  |  |
