# Supplementary material for: Human Perceptions Mirror Realities of Carnivore Attack Risk for Livestock: Implications for Mitigating Human-Carnivore Conflict
Source: PLoS One. 2016 Sep 12;11(9):e0162685. doi: 10.1371/journal.pone.0162685 (PMC5019480; doi:10.1371/journal.pone.0162685)
Supplement: S2 Table — (DOCX) [file pone.0162685.s005.docx]

**Table S2.** Statistics from ordinal logistic regression models testing whether the percent land-use within 4 km of villages influenced owners' perceptions of carnivore risk. Statistics include the coefficient or intercept value, standard error (SE), t-value, *P*-value and the model residual deviance, Akaike Information Criterion (AIC) and chai-square p-value (χ^2^). χ^2^ > 0.05 indicate strong model fit.

| Carnivore species | Land-use | Statistic | Value | SE | t-value | *P*-value | Residual deviance | AIC | χ^2^ |
| --- | --- | --- | --- | --- | --- | --- | --- | --- | --- |
| Tiger | village | intercept 1\|2 | 0.36 | 0.72 | 0.49 | 0.62 | 38.39 | 46.39 | 7.91E-04 |
|  |  | intercept 2\|3 | 0.57 | 0.73 | 0.78 | 0.43 |  |  |  |
|  |  | intercept 3\|4 | 3.17 | 1.20 | 2.63 | 0.01 |  |  |  |
|  |  | coefficient | 0.49 | 1.09 | 0.45 | 0.65 |  |  |  |
|  | agricultural field | intercept 2\|3 | 0.29 | 0.71 | 0.40 | 0.69 | 69.26 | 75.26 | 7.13E-04 |
|  |  | intercept 3\|4 | 2.51 | 0.88 | 2.87 | 0.00 |  |  |  |
|  |  | coefficient | 0.00 | 0.02 | 0.05 | 0.96 |  |  |  |
|  | agricultural field-forest edge | intercept 2\|3 | -1.83 | 0.95 | -1.93 | 0.05 | 66.74 | 72.74 | 3.79E-02 |
|  |  | intercept 3\|4 | 2.85 | 0.91 | 3.12 | 0.00 |  |  |  |
|  |  | coefficient | 0.14 | 0.07 | 2.10 | 0.04 |  |  |  |
|  | forest | intercept 1\|2 | -3.56 | 1.27 | -2.80 | 0.01 | 58.29 | 64.29 | 9.98E-01 |
|  |  | intercept 3\|4 | -2.25 | 1.13 | -2.00 | 0.05 |  |  |  |
|  |  | coefficient | 0.01 | 0.02 | 0.26 | 0.79 |  |  |  |
| Leopard | village | intercept 1\|2 | -0.78 | 0.41 | -1.90 | 0.06 | 97.66 | 105.66 | 9.97E-07 |
|  |  | intercept 2\|3 | -0.57 | 0.40 | -1.43 | 0.15 |  |  |  |
|  |  | intercept 3\|4 | 0.01 | 0.39 | 0.01 | 0.99 |  |  |  |
|  |  | coefficient | 0.15 | 0.38 | 0.39 | 0.70 |  |  |  |
|  | agricultural field | intercept 1\|2 | 1.51 | 0.69 | 2.20 | 0.03 | 111.96 | 119.96 | 2.01E-07 |
|  |  | intercept 2\|3 | -3.55 | 1.15 | -3.09 | 0.00 |  |  |  |
|  |  | intercept 3\|4 | -0.60 | 0.65 | -0.91 | 0.36 |  |  |  |
|  |  | coefficient | 0.01 | 0.02 | 0.60 | 0.55 |  |  |  |
|  | agricultural field-forest edge | intercept 2\|3 | -2.87 | 0.96 | -2.98 | 0.00 | 66.11 | 72.11 | 2.28E-03 |
|  |  | intercept 3\|4 | -0.97 | 0.81 | -1.20 | 0.23 |  |  |  |
|  |  | coefficient | -0.03 | 0.07 | -0.48 | 0.63 |  |  |  |
|  | forest | intercept 1\|2 | -2.18 | 0.77 | -2.84 | 0.00 | 144.29 | 152.29 | 1.00E-09 |
|  |  | intercept 2\|3 | -0.72 | 0.66 | -1.09 | 0.27 |  |  |  |
|  |  | intercept 3\|4 | 0.74 | 0.65 | 1.14 | 0.25 |  |  |  |
|  |  | coefficient | 0.01 | 0.01 | 0.80 | 0.43 |  |  |  |
