# Supplementary material for: Human Perceptions Mirror Realities of Carnivore Attack Risk for Livestock: Implications for Mitigating Human-Carnivore Conflict
Source: PLoS One. 2016 Sep 12;11(9):e0162685. doi: 10.1371/journal.pone.0162685 (PMC5019480; doi:10.1371/journal.pone.0162685)
Supplement: S3 Table — (DOCX) [file pone.0162685.s006.docx]

**S3 Table.** Statistics from ordinal logistic regression models testing whether the mean observed risk within 500 m of villages influenced owners' perceptions of carnivore risk. Statistics include the coefficient or intercept value, standard error (SE), t-value, *P*-value and the model residual deviance, Akaike Information Criterion (AIC) and chai-square p-value (χ^2^). χ^2^ > 0.05 indicate strong model fit.

| Carnivore species | Land-use | Statistic | Value | SE | t-value | *P*-value | Residual deviance | AIC | χ^2^ |
| --- | --- | --- | --- | --- | --- | --- | --- | --- | --- |
| Tiger | village | intercept 1\|2 | -2.52 | 1.15 | -2.19 | 0.03 | 38.10 | 46.10 | 8.72E-04 |
|  |  | intercept 2\|3 | 0.08 | 0.75 | 0.11 | 0.91 |  |  |  |
|  |  | intercept 3\|4 | 0.30 | 0.75 | 0.40 | 0.69 |  |  |  |
|  |  | coefficient | 3.59 | 5.33 | 0.67 | 0.50 |  |  |  |
|  | agricultural field | intercept 1\|2 | -2.79 | 0.74 | -3.80 | 0.00 | 67.39 | 73.39 | 8.14E-04 |
|  |  | intercept 2\|3 | -0.52 | 0.49 | -1.05 | 0.29 |  |  |  |
|  |  | coefficient | -2.88 | 3.45 | -0.83 | 0.40 |  |  |  |
|  | agricultural field-forest edge | intercept 1\|2 | -1.48 | 0.48 | -3.10 | 0.00 | 69.79 | 75.79 | 1.71E-02 |
|  |  | intercept 2\|3 | 2.92 | 0.76 | 3.83 | 0.00 |  |  |  |
|  |  | coefficient | -3.48 | 3.49 | -1.00 | 0.32 |  |  |  |
|  | forest | intercept 1\|2 | 2.41 | 0.58 | 4.12 | 3.72E-05 | 58.00 | 64.00 | 9.96E-01 |
|  |  | intercept 2\|3 | 3.72 | 0.83 | 4.45 | 8.46E-06 |  |  |  |
|  |  | coefficient | -1.16 | 5.11 | -0.23 | 8.21E-01 |  |  |  |
| Leopard | village | intercept 1\|2 | 0.19 | 0.60 | 0.32 | 0.75 | 96.42 | 104.42 | 9.13E-07 |
|  |  | intercept 2\|3 | 0.77 | 0.61 | 1.27 | 0.20 |  |  |  |
|  |  | intercept 3\|4 | 0.98 | 0.61 | 1.60 | 0.11 |  |  |  |
|  |  | coefficient | 0.23 | 0.81 | 0.28 | 0.78 |  |  |  |
|  | agricultural field | intercept 1\|2 | -1.09 | 0.44 | -2.48 | 0.01 | 110.82 | 118.82 | 1.79E-07 |
|  |  | intercept 2\|3 | 0.96 | 0.43 | 2.22 | 0.03 |  |  |  |
|  |  | intercept 3\|4 | 3.91 | 1.06 | 3.71 | 0.00 |  |  |  |
|  |  | coefficient | 0.47 | 3.39 | 0.14 | 0.89 |  |  |  |
|  | agricultural field-forest edge | intercept 1\|2 | 0.67 | 0.48 | 1.40 | 0.16 | 62.87 | 68.87 | 2.64E-03 |
|  |  | intercept 2\|3 | 2.47 | 0.69 | 3.59 | 0.00 |  |  |  |
|  |  | coefficient | 0.18 | 3.59 | 0.05 | 0.96 |  |  |  |
|  | forest | intercept 1\|2 | -1.26 | 0.59 | -2.14 | 0.03 | 110.77 | 118.77 | 1.82E-07 |
|  |  | intercept 2\|3 | 0.79 | 0.57 | 1.38 | 0.17 |  |  |  |
|  |  | intercept 3\|4 | 3.74 | 1.12 | 3.33 | 0.00 |  |  |  |
|  |  | coefficient | -0.20 | 0.75 | -0.27 | 0.79 |  |  |  |
