# Supplementary material for: Human Perceptions Mirror Realities of Carnivore Attack Risk for Livestock: Implications for Mitigating Human-Carnivore Conflict
Source: PLoS One. 2016 Sep 12;11(9):e0162685. doi: 10.1371/journal.pone.0162685 (PMC5019480; doi:10.1371/journal.pone.0162685)
Supplement: S4 Table — (DOCX) [file pone.0162685.s007.docx]

**S4 Table.** Statistics from ANOVAs testing whether an owner’s previous experience with livestock depredation (‘yes’ or ‘no’) affected perceptions of carnivore risk. P > 0.05 indicates no effect of previous experience on perceived risk.

| Carnivore | Land-use | Degrees of freedom | F-value | *P*-value |
| --- | --- | --- | --- | --- |
| Tiger | Village | 1, 12 | 2.545 | 0.137 |
|  | Field | 1, 28 | 0.415 | 0.524 |
|  | Field-forest edge | 1, 30 | 2.748 | 0.105 |
|  | Forest | 1, 80 | 2.713 | 0.103 |
| Leopard | Village | 1, 34 | 0.437 | 0.513 |
|  | Field | 1, 40 | 0.647 | 0.426 |
|  | Field-forest edge | 1, 27 | 0.002 | 0.964 |
|  | Forest | 1, 45 | 0.000 | 0.984 |
