# Supplementary material for: Human Perceptions Mirror Realities of Carnivore Attack Risk for Livestock: Implications for Mitigating Human-Carnivore Conflict
Source: PLoS One. 2016 Sep 12;11(9):e0162685. doi: 10.1371/journal.pone.0162685 (PMC5019480; doi:10.1371/journal.pone.0162685)
Supplement: S5 Table — (DOCX) [file pone.0162685.s008.docx]

**S5 Table.** Statistics from ANOVAs testing whether an owner’s previous experience with livestock depredation (‘use’ or ‘no use’) affected future use of livestock protection methods.

P > 0.05 indicates no effect of previous experience on perceived risk.

| Livestock protection method | Degrees of freedom | F-value | *P*-value |
| --- | --- | --- | --- |
| Change grazing area | 1, 93 | 5.176 | 0.025 |
| Strengthen livestock enclosure | 1, 93 | 0.255 | 0.615 |
| Hire herder to protect grazing livestock | 1, 93 | 0.670 | 0.415 |
| Use family member to protect grazing livestock | 1, 93 | 0.122 | 0.727 |
| Tie livestock near house | 1, 93 | 0.390 | 0.534 |
| Be more alert | 1, 77 | 0.393 | 0.533 |
